# Dietary Habits and Cooking Methods Could Reduce Avoidable Exposure to PCBs in Maternal and Cord Sera

**DOI:** 10.1038/s41598-017-17656-9

**Published:** 2017-12-11

**Authors:** Weiwei Jin, Masae Otake, Akifumi Eguchi, Kenichi Sakurai, Hiroko Nakaoka, Masahiro Watanabe, Emiko Todaka, Chisato Mori

**Affiliations:** 1 Center for Preventive Medical Sciences, Chiba University, Chiba, Japan; 2 Department of Bioenvironmental Medicine, Graduate School of Medicine, Chiba University, Chiba, Japan

## Abstract

Polychlorinated biphenyls (PCBs), like other persistent organic pollutants, are accumulating throughout the food chain and pose health threats to humans, especially children and foetuses. There is no protocol for reducing the contamination levels of the PCBs in humans. This study identified food items and cooking methods that reduce serum PCB levels by analysing data collected from the Chiba Study of Mother and Child Health. The sample size was 194 subjects. Serum PCB levels were measured using gas chromatography–electron capture negative ionization quadrupole mass spectrometry. Information on dietary habits was obtained from a brief diet history questionnaire that included questions about food items and cooking methods. Food items were categorized into food groups, and nutrient levels were calculated based on food item consumption. Principal component analysis and lasso regression were used as statistical methods. The analyses of food items and nutrients suggested that food items rich in dietary fibre reduce avoidable exposure to PCBs, as could grilling and deep frying of food, which could reduce avoidable exposure to serum PCBs in mothers and foetuses. (174 words).

## Introduction

Previous studies have shown that virtually every person on this planet has been exposed to persistent organic pollutants (POPs) via different intermediaries (e.g., air, water, and food)^1–4^. POPs tend to accumulate in the fatty tissues of our bodies because of their high chemical stability, persistence, and pronounced lipophilicity. These factors make it difficult to reduce contamination levels^5–7^. The health threats posed by POPs to humans have been investigated in several cohort studies worldwide^8–12^. These studies have revealed that exposure to one of the more common types of POP, polychlorinated biphenyls (PCBs), increases the risk of cancer^13–15^ and causes liver dysfunction, skin lesions (chloracne), and nervous system abnormalities^8^. PCBs also affect the immune system^16,17^ and increase mortality rates from cardiovascular disease^9^. Foetuses and children are more susceptible to these environmental contaminants than adults^1^. Studies show that children exposed to PCBs experience poor cognitive function in early childhood^10,11,18^. Furthermore, exposure to high levels of PCBs during the foetal period leads to a low birth weight in infants^19–22^. Therefore, reducing the contamination level of PCBs in humans could significantly improve the health of future generations.

The food chain is the primary source of POP bioaccumulation^5^. Previous studies have shown that fish and meat (e.g., beef, pork, and chicken) are the main food sources of POP exposure (e.g., PCBs)^23–25^, despite the essential nutrients provided by these foods^26^. It has also been shown that the contamination levels of POPs differ in different food items^23,27,28^. Furthermore, cooking methods may reduce PCB levels in different food items^29,30^.

Previous studies have tested various interventions to reduce PCB levels in humans, such as drug treatment and dietary methods. Studies have indicated that drugs such as colestimide reduce blood dioxin levels (i.e., polychloro-dibenzon-p-dioxin, polychloro-dibenzofuran, and coplanar polychlorinated benzene)^31,32^. However, colestimide produces numerous side effects (e.g., gastrointestinal bleeding and vitamin and calcium deficiency). A recent study also showed that colestimide may not effectively reduce serum PCB levels in Yusho patients (people who were exposed to rice oil contaminated with PCBs and dioxin-like compounds in 1968)^33^. Attempts have also been made to reduce the contamination levels of POPs through dietary methods. Fermented brown rice with *Aspergillus oryzae* ^34^ and dietary olestra^34^ may reduce PCB levels in the body. Additionally, cooking processes can reduce the concentration of organic environmental pollutants (e.g., PCBs) in food items^29,30^. Nevertheless, appropriate guidelines for reducing POP contamination in humans have yet to be established, despite the efforts made in these studies.

The present study examined the relationship between dietary habits, based on a comparatively low-cost data transfer method involving a brief diet history questionnaire (BDHQ), and serum PCB levels in maternal and cord blood from pregnant women in Japan according to the measures proposed in a recent report^35^. The data analysed in this study were obtained from the Chiba Study of Mother and Child Health (C-MACH)^36^, which is a birth cohort study based on the developmental origins of health and disease hypothesis, performed in pregnant women who enrolled at less than 13 weeks of gestation. We analysed the relationship between serum PCB levels and food groups (following^37^). We then investigated the relationship between serum PCB levels and different nutrients, calculated based on the consumption of food items. We also examined the association between serum PCB levels and different cooking methods. This study determined an effective intervention method for reducing serum PCB levels.

## Materials and Methods

### Set-up

Dietary data from the BDHQ and data on serum PCB levels were obtained from the Onodera Ladies Clinic and Yamaguchi Women’s Hospital in Japan. The recruitment period was February 2014 to June 2015. The participants in this study were pregnant women at <13 weeks of gestation and their unborn children. We will continue with follow-up surveys until the children reach 5 years of age. The data used in this study were collected during the last trimester of pregnancy. Stillbirths and data from mother and child pairs or children of women who withdrew their consent were excluded, as were data from women who transferred to another hospital during the data collection period. The Biomedical Research Ethics Committee of the Graduate School of Medicine of Chiba University approved this study (ID: 759), which was performed in accordance with the approved guidelines and regulations of the Declaration of Helsinki. Written informed consent was obtained from each participant.

A total of 289 samples were initially collected from the Onodera Ladies Clinic and Yamaguchi Women’s Hospital. Some samples were removed because key information was missing (i.e., the mother’s body mass index (BMI), age, parity, BDHQ, or PCB levels in maternal serum), which reduced the sample size to 202. The sample size was further reduced to 194 after excluding mothers who consumed alcohol during pregnancy. The final analysed sample size for investigating the association of PCB levels in maternal and cord serum with dietary habits and cooking methods was 189 after the removal of samples with missing data on PCB levels in cord serum.

### Data acquisition

The mothers’ age and parity were obtained from the questionnaires at 12 weeks of gestation, and their BMIs were calculated based on the physical information acquired from the questionnaires. The mothers’ dietary habits were recorded using the BDHQ during the last trimester, including information about both food items and cooking methods. The contamination levels of serum PCBs were assessed in maternal blood samples collected at an average of 35 weeks of gestation, with a range of 30 to 39 weeks of gestation (Onodera Ladies Clinic: average: 32 weeks of gestation, range: 30 to 36 weeks of gestation; Yamaguchi Women’s Hospital: average: 36 weeks of gestation, range: 34 to 39 weeks of gestation) and cord blood. PCB levels in the serum samples from maternal and cord blood were analysed using gas chromatography–electron capture negative ionization quadrupole mass spectrometry (GC-NICI-qMS)^38^.

The serum samples (0.3–0.4 g) were desaturated using 1 M potassium hydroxide/MeOH (1 mL) in the GC-NICI-qMS analysis. Target compounds were extracted twice with n-hexane (500 μL), and the samples were spiked with PCB 23, 30, 55, and 207 (40 pg each) as surrogate internal standards. The two extracts were combined and washed with ultrapure water. The remaining residue after evaporation of the solvent was dissolved in n-hexane (3 mL), passed through a glass column packed with 44% sulfuric acid silica gel (500 mg), and concentrated until almost dry. PBB 154 (20 pg) dissolved in decane (200 μL) was added as a syringe spike.

The instrument used for the analyses was a JMS-Q1050GC (JEOL Ltd., Tokyo, Japan) quadrupole mass spectrometer equipped with an Agilent 7890B gas chromatograph and a 7693 autosampler (Agilent Technologies Inc., Tokyo, Japan). An HP5-MSUI fused-silica capillary column (30 m × 0.25 mm ID × 0.25 μm film Agilent Technologies Inc., Tokyo, Japan) was employed for GC separation. The injector was operated in pulsed splitless mode at 280 °C. Helium (Purity: >99.99995) was used as the GC carrier gas at a constant flow rate of 1.3 mL/min, and methane (Purity: >99.999) was the reagent gas for the NICI source. The identification and quantification of the 24 PCB congeners were achieved via monitoring of chlorine ions [Cl^−^: m/z: 35] through selected ion monitoring (SIM) analysis using a NICI-MS detector.

The samples employed in this study were QA/AC samples collected from a PCB measurement campaign of performed in our laboratory^36^, which is accredited in compliance with ISO/IEC 17025:2005 standards (Accreditation of Certification Body: Japan Accreditation Board). The detection limits for individual PCBs using this method were 1.9–20 pg g^−1^ wet wt. An inter-calibration exercise organized by the NIST using Standard Reference Material 1957^39^ was applied to ensure the quality and analyses of PCBs.

### Statistical analysis

Dietary data were re-sorted into food groups based on a previous study prior to the primary analysis^37^. This organization reduced the number of parameters in the analysis. The nutrient contents of the food items were calculated based on the consumption amounts indicated in the BDHQ, to further investigate the association between serum PCB levels and dietary habits^40^.

The main analysis tool used in this study was R ver. 3.3.3^41^. A principal component analysis (PCA) using the FactoMineR package^42^ was combined with lasso regression^43^ to analyse the relationship between serum PCB levels and different food groups. The lasso regression was used to analyse the relationship between serum PCB levels and nutrients and that between serum PCB levels and cooking methods. Some of the mothers’ characteristics (e.g., parity, BMI, and age)^44^ were also included in the analyses of the relationships between dietary habits and serum PCB levels because of the high correlation between serum PCB levels and these indices.

## Results

### Participant characteristics

Table 1 presents the maternal demographics and lifestyle characteristics employed for the analyses in this study (n = 194), including age, BMI, parity, marital status, infertility treatment, smoking history, and education. Table 1 shows that 93.81% of the participants were over 25 years old and that 75.26% had a BMI between 18.5 and 24.9. Most of the participants (98.97%) were married; 80.41% exhibited an education level above high school; and 80.93% were non-smokers. Additionally, 89.18% of the participants had received no infertility treatment, and 40.21% of the participants were nulliparous.

**Table 1:** Maternal characteristics in the analysed sample.

Variables	Number	Percentage (%)
Total Sample Size	194	
Mothers’ age		
<25	12	6.19
25–29	29	14.59
30–34	86	44.33
≥35	67	34.54
Mothers’ BMI		
<18.5	35	18.04
18.5–24.9	146	75.26
≥25	13	6.70
Parity		
0	78	40.21
1	116	59.79
Marital Status		
Married	192	98.97
Divorced	1	0.52
Never Married	1	0.52
Infertility Treatment		
Yes	16	8.25
No	173	89.18
Data missing	5	2.58
Smoking History		
Smoker	2	1.03
Ex-smoker	34	17.53
Non-smoker	157	80.93
Data missing	1	0.52
Education		
Junior-high School	5	2.58
High School	33	17.01
Vocational College, Junior College, Technical College	78	40.21
University, Graduate School	77	39.69
Data missing	1	0.52

### Analyses of PCB levels

Table 2 provides a summary of the analyses of PCB levels in maternal and cord sera. PCB concentrations in cord serum were highly correlated with those in maternal serum. Twenty-four PCB congeners were identified in this study; CB28, CB60, CB66, CB87, CB178, CB199, CB206, and CB209 were omitted from the summary (Table 2) because of a low detection rate in the samples (less than 50% in maternal serum and less than 25% in cord serum). The median concentrations of total PCBs in the maternal and cord sera were 0.37 and 0.10 ng g^−1^ wet wt., respectively. The maximum concentration of total PCBs in maternal and cord sera were 1.48 and 0.51 ng g^−1^ wet wt., respectively. The dominant congeners of the PCBs in the maternal and cord blood sera were CB138, CB153, and CB180, which is consistent with the results of previous studies^45–53^. The concentration of PCBs detected in the blood in this study was lower than in previous studies^45–47,51,53–55^. The reason for this difference may be that the subjects of this study were young perinatal women, in whom serum PCB concentrations either may be low due to their young age or may be lowered as a result of pregnancy^56^.

**Table 2:** Distribution of PCB congeners in maternal and cord sera (LOD: limit of detection).

	Maternal Blood					Cord Blood				
	Minimum	25^th^ percentile	Median	75^th^ percentile	Maximum	Minimum	25^th^ percentile	Median	75^th^ percentile	Maximum
PCBs (ng g-1 wet wt.)										
CB74	LOD	0.0054	0.0090	0.0145	0.0402	LOD	LOD	0.0032	0.0070	0.0278
CB99	LOD	0.0102	0.0148	0.0216	0.0784	LOD	LOD	LOD	0.0094	0.0306
CB105	LOD	0.0003	0.0031	0.0068	0.0397	LOD	LOD	LOD	0.0032	0.0314
CB118	0.0025	0.0152	0.0225	0.0332	0.0933	LOD	LOD	0.0037	0.0102	0.0437
CB126	LOD	0.0027	0.0041	0.0054	0.0164	LOD	LOD	LOD	0.0013	0.0108
CB138	0.0069	0.0486	0.0676	0.0913	0.2528	LOD	0.0110	0.0156	0.0233	0.0763
CB146	0.0003	0.0118	0.0154	0.0211	0.0698	LOD	LOD	0.0043	0.0067	0.0244
CB153	0.0094	0.0698	0.0949	0.1351	0.4099	0.0013	0.0134	0.0186	0.0266	0.1056
CB156	LOD	0.0065	0.0095	0.0131	0.0386	LOD	LOD	0.0015	0.0024	0.0119
CB170	0.0003	0.0189	0.0246	0.0323	0.0846	LOD	0.0028	0.0041	0.0060	0.0245
CB177	LOD	0.0050	0.0077	0.0109	0.0232	LOD	LOD	LOD	0.0027	0.0146
CB180	0.0050	0.0399	0.0522	0.0729	0.2213	0.0009	0.0070	0.0096	0.0136	0.0587
CB183	LOD	0.0035	0.0054	0.0095	0.0331	LOD	LOD	LOD	0.0018	0.0109
CB187	0.0042	0.0202	0.0262	0.0385	0.1163	LOD	0.0034	0.0047	0.0066	0.0345
CB194	0.0002	0.0065	0.0089	0.0119	0.0323	LOD	LOD	0.0011	0.0019	0.0091
CB201	0.0013	0.0072	0.0094	0.0125	0.0346	LOD	0.0011	0.0018	0.0026	0.0109
Total	0.0426	0.2918	0.3708	0.5287	1.4764	0.0107	0.0600	0.0906	0.1259	0.5065

**Figure 1:**
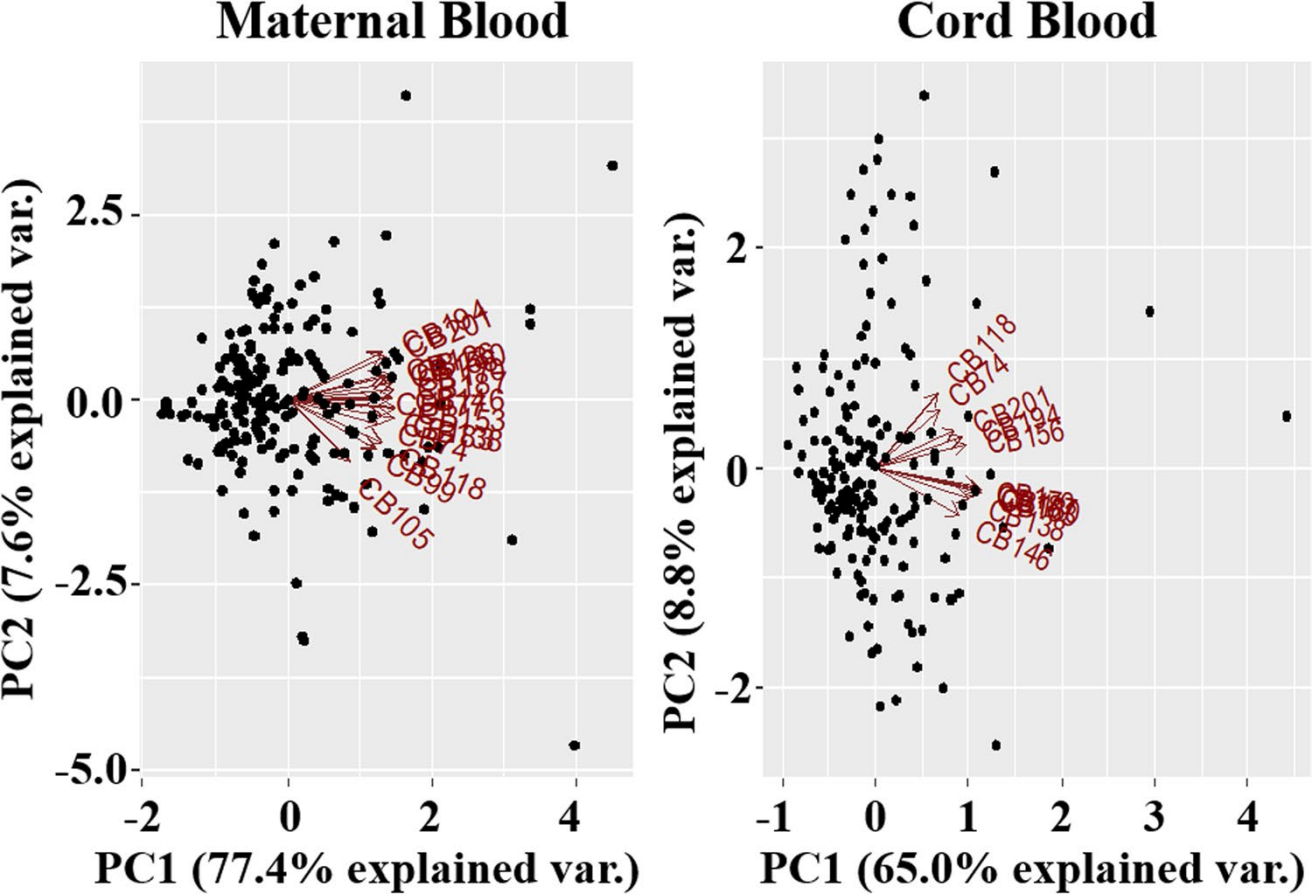
PCA for various PCB congeners measured in maternal blood during the last trimester and cord blood (PC1 vs. PC2). Sixteen PCB congeners (CB74, CB99, CB105, CB118, CB126, CB138, CB146, CB153, CB156, CB170, CB177, CB180, CB183, CB187, CB194, and CB201) are shown for maternal blood; eleven PCB congeners (CB74, CB118, CB138, CB146, CB153, CB156, CB170, CB180, CB187, CB194, CB201) are shown for cord blood.

PCA of the different congeners of PCBs measured in maternal and cord sera was also performed (Fig. 1). CB99, CB105, CB126, CB177, and CB183 were further excluded from the analyses of PCBs in cord serum because of their low concentrations (median < limitation of detection) (Table 2). The results show that the dispersion of different congeners of PCBs was low (aligned in the same direction of PC1) in maternal and cord blood sera. Therefore, we used the total PCBs for the main analysis in this study.

### Main results

Table 3 presents the results of the association analysis between maternal serum PCB levels and the consumed food groups, analysed using the combination of PCA and lasso regression. Parity was negatively related to PCB levels in maternal and cord sera, and the mother’s BMI and age showed a positive relation, which corresponds to previous studies^44,57–59^. The top three explained variant components (PC1, PC2, and PC3) were positively related to PCB levels in maternal and cord sera. Comparison of the association between each principal component and PCB levels in maternal serum revealed that the association was weaker in cord serum.

**Table 3:** Combination of PCA and lasso regression for food groups and PCB levels in maternal and cord sera (adjusted by parity, mothers’ BMI, and mothers’ age).

	Maternal Blood	Cord Blood
(Intercept)	0.0398	0.0640
PC1	0.0101	0.0034
PC2	0.0055	0.0025
PC3	0.0026	0.0017
PC4	—	0.0072
PC5	0.0099	0.0024
PC7	0.0028	0.0052
PC8	—	0.0016
PC9	—	−0.0038
PC11	—	−0.0069
PC12	—	−0.0024
PC13	—	0.0077
PC14	—	0.0061
PC15	−0.0113	−0.0057

**Figure 2:**
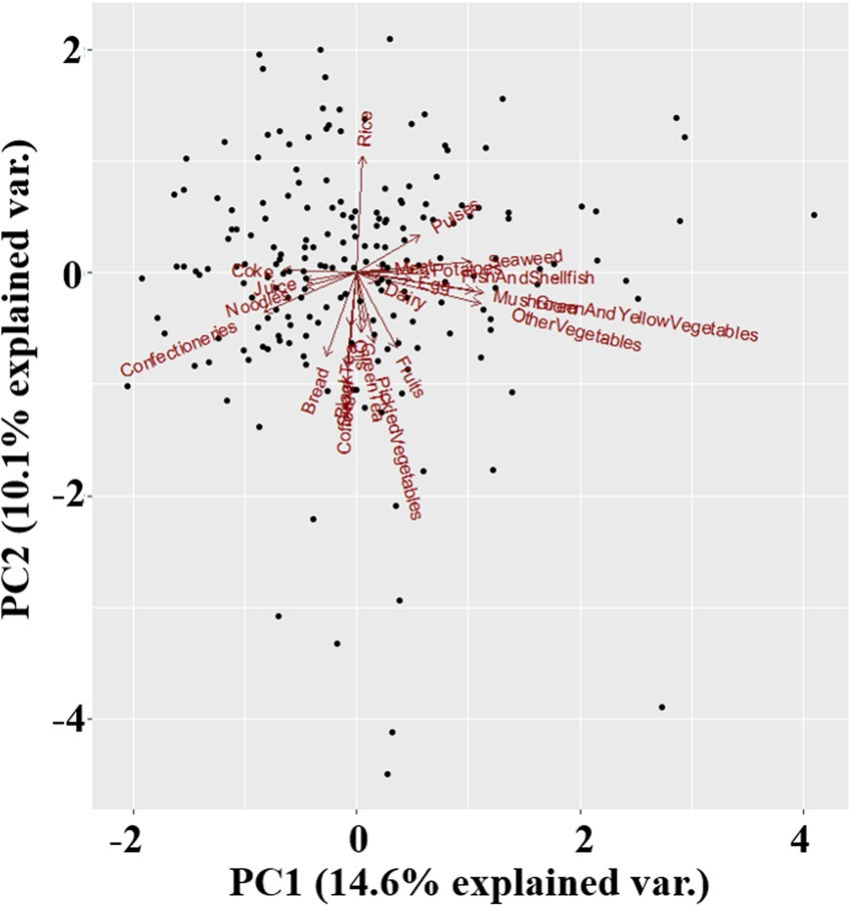
PCA for different food groups and serum PCB levels (PC1 vs. PC2). Twenty-two food groups (dairy, meat, fish and shellfish, eggs, pulses, potatoes, pickled vegetables, green and yellow vegetables, other vegetables, mushroom, seaweed, confectioneries, fruits, oils, bread, noodles, green tea, black tea, coffee, coke, juice, sugar, and rice) are shown.

**Figure 3:**
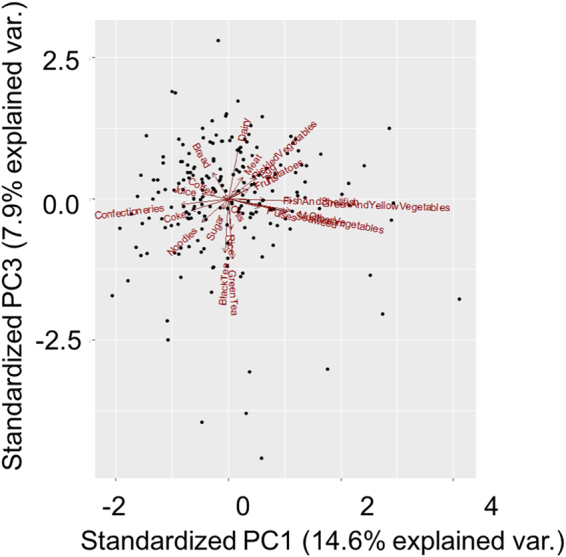
PCA for different food groups and serum PCB levels (PC1 vs. PC3). Twenty-two food groups (dairy, meat, fish and shellfish, eggs, pulses, potatoes, pickled vegetables, green and yellow vegetables, other vegetables, mushroom, seaweed, confectioneries, fruits, oils, bread, noodles, green tea, black tea, coffee, coke, juice, sugar, and rice) are shown.

Figures 2 and 3 illustrate the correlations between different food groups and the relationship between serum PCB levels and food groups (PC1 vs. PC2 and PC1 vs. PC3, respectively). Figure 2 shows that fish/shellfish, seaweed, mushrooms, and vegetables (Group 1) were correlated with each other, as were meat, potatoes, eggs, and dairy products (Group 2). Group 3 included cola, juice, noodles, and confectionery. There also appeared to be a correlation between fruits, pickled vegetables, green tea, black tea, coffee, and bread (Group 4). Rice was on the opposite side of Group 4. Figure 3 shows the correlation between fish/shellfish, green and yellow vegetables, other vegetables (not including green and yellow vegetables), seaweed, and pulses (Group 5). Confectionery, coffee, juice, and cola appeared on the opposite side of Group 5. Meat, pickled vegetables, potatoes, and fruits (Group 6) exhibited a correlation, with noodles on the opposite side. Rice, green tea, and black tea (Group 7) also appeared to correlate with each other, and bread was on the opposite side of Group 7.

The results for PC1 indicated that foods in Group 1 and Group 2 positively contributed to the elevation of serum PCB levels, and foods in Group 3 presented a negative contribution. The results for PC2 suggested that rice positively contributed to the elevation of serum PCB levels, and foods in Group 4 made a negative contribution. The results for PC3 revealed that foods in Group 6 (dairy products and bread) made a positive contribution to the elevation of serum PCB levels, and foods in Group 7 (vegetables other than green and yellow vegetables, seaweed, and pulses) made a negative contribution. These correlations between different food items suggest that vegetables, fruits, mushrooms, rice, tea, seaweed, and pulses, which contain a comparatively high amount of dietary fibre, are negatively associated with an increase in serum PCB levels.

A lasso regression was performed to investigate the association between nutrients and PCB levels in maternal and cord sera (Table 4). The regression showed that most of the nutrients were excluded from the analyses of PCB levels in maternal serum because of a low correlation. The nutrients that exhibited a comparatively strong negative association with serum PCB levels included manganese, pantothenic acid, saturated fatty acid, and total dietary fibre. However, the difference in the distribution of manganese, pantothenic acid, and saturated fatty acid between different food items was quite small, and the amount of dietary fibre was high in food items such as vegetables, tea, rice, and pulses^60^. A similar observation was made in the analysis of the association between nutrients and PCB levels in cord serum.

**Table 4:** Lasso regression for nutrients and PCB levels in maternal and cord sera (adjusted by parity, mothers’ BMI, and mothers’ age).

	Maternal Blood	Cord Blood
(Intercept)	−0.1307	−0.0986
Carbohydrate	0.0002	0.0009
Sodium	>−0.0000	—
Calcium	>−0.0000	—
Magnesium	>−0.0000	−0.0006
Iron	—	0.0004
Zinc	—	−0.0187
Copper	—	−0.0005
Manganese	−0.0630	−0.0175
Beta Carotene Equivalent	>−0.0000	>−0.0000
Retinol Equivalent	<0.0000	>−0.0000
Vitamin D	—	0.0035
Alpha Tocopherol	0.0454	0.0488
Vitamin B1	0.0842	0.0835
Vitamin B2	—	−0.1413
Niacin	—	−0.0022
Vitamin B6	—	0.1210
Vitamin B12	—	−0.0052
Folic Acid	—	0.0006
Pantothenic Acid	−0.0392	0.0328
Vitamin C	—	−0.0010
Saturated Fatty Acid	−0.0126	—
Monounsaturated Fatty Acid	—	−0.0077
Polyunsaturated Fatty Acid	—	−0.0014
Cholesterol	—	0.0002
Total Dietary Fibre	−0.0132	−0.0176
Salt Equivalent	—	0.0046

We further analysed the relationship between cooking methods and PCB levels in maternal and cord sera using lasso regression (Table 5). The results revealed that grilling of fish and meat was negatively associated with serum PCB levels. Deep frying of food items other than fish was also negatively associated with serum PCB levels. Grilled meat exerted a greater negative effect on serum PCB levels than grilled and deep-fried fish. The association between PCB levels in cord sera and cooking methods was weaker overall than that for maternal sera because of the low PCB concentration in cord sera.

**Table 5:** Lasso regression of cooking methods and PCB levels in maternal and cord sera (adjusted by parity, mothers’ BMI, and mothers’ age).

	Maternal Blood	Cord Blood
(Intercept)	−0.1373	0.0867
Fish		
Raw	0.0038	0.0010
Grilling	−0.0003	—
Stew	0.0003	<0.0000
Deep Frying	0.0015	—
Other than fish		
Grilling (Meat)	−0.0037	−0.0004
Pan Frying (Hamburger Meat)	0.0014	0.0001
Deep Frying	−0.0010	—
Sautéed	0.0007	—
Stew	0.0004	<0.0000

## Discussion

Dietary habits are closely associated with the contamination levels of PCBs in humans because exposure to PCBs primarily occurs via the food chain^4,5^, and some food items and cooking processes reduce avoidable exposure to PCBs (Figs 2 and 3, and Table 5). Therefore, we investigated the relationship between PCB contamination levels and different dietary habits and cooking methods. The results presented in Figs 2 and 3 suggested that people who consumed fish, meat, and vegetables tended to eat less junk food. People who frequently consumed junk food tended to eat bread as a staple food rather than rice. Overall, the results show that people who ate regular meals (e.g., consumed foods such as fish, meat, vegetables, rice, and bread) tended to exhibit comparatively high contamination levels of PCBs. This tendency occurred because food items such as fish, meat, eggs, and dairy products are the main food sources of PCB exposure^23–25,27,61,62^. However, the results also indicated that foods such as vegetables, fruits, mushrooms, rice, tea, seaweed, and pulses reduced avoidable exposure to PCBs in maternal and cord sera. Analyses of the associations between nutrients and serum PCB levels revealed that nutrients were distributed comparatively equally among different food items, and dietary fibre reduced avoidable exposure to PCBs. High contents of dietary fibre are found in food items such as vegetables, fruits, seaweed, rice, nuts, pulses, and tea^60^, which is consistent with the results shown in Figs 2 and 3. Therefore, the consumption of food items that contain a high content of dietary fibre may reduce avoidable exposure to PCBs, as suggested in previous studies^63–67^.

Previous studies have suggested that cooking reduces PCB levels in food^26,68^, and the reduction of PCB contamination by cooking depends on the particular food item and the applied cooking method. A general reduction of fatty tissue in food items may decrease the contamination levels of PCBs because of the lipophilic properties of PCBs^29,30^. The present study showed that grilling and deep frying were associated with lower serum levels of PCBs. Grilling reduces the contamination level of PCBs due to the loss of fatty tissue during the cooking process, and deep frying extracts PCBs into the cooking oil. However, grilling fish was not as effective as grilling meat in lowering serum levels of PCBs because of the comparatively high concentration of PCBs in fish^15^ (Table 5). Previous studies have indicated that deep frying is the most effective cooking method for reducing PCB levels in food^69^, but we found that deep frying was not as effective as grilling. We may have obtained this result because of the particular cooking methods used by the participants in this study. For example, grilling was employed to cook fish and meat, and deep frying was used to cook meat and vegetables. Therefore, the effect of PCB reduction on vegetables could have been low because the contamination level in vegetables was low^27^. Another reason may be the changes in the texture of the food associated with different cooking methods. For example, any burnt areas on food may obstruct the absorption of PCBs during digestion.

Other studies have also shown that the concentration of PCBs is correlated with the concentration of other POPs, such as organohalogen pesticides, polybrominated diphenyl ethers, PCDDs, and PCDFs^28,54^. Reduction interventions to avoid PCB exposure may also be used for these POPs because most of these POPs exhibit lipophilic properties similar to PCBs. The analysis of serum PCB levels revealed that the correlation between maternal serum PCB levels and cord serum PCB levels was high (Table 2). PCB contamination in cord serum is related to the health of the foetus. Therefore, a lower maternal level of PCBs may reduce PCB contamination levels in foetuses. However, the data employed in this study were acquired from pregnant women in Japan, and dietary habits are likely different in other countries. People in countries and regions near the sea, such as Japan, tend to consume more fish than meat. However, people living in inland countries and regions tend to consume more meat. Therefore, the results may differ if the analysed data were to be collected from an inland country or region. Different dietary habits in different countries and regions should be considered in future studies.

This study has some limitations. First, the sample size was comparatively small (n = 194). Therefore, a cohort study with a larger sample size should be performed in the future. Second, crossover analyses involving the consumption of different food items and use of different cooking methods were not performed in this study. A case-control study of this nature based on human subjects may result in an even smaller sample size and increase the likelihood of bias. Furthermore, a case-control study with animals may not reflect the situation in humans because of the differences in human and animal metabolism. Finally, the data on cooking methods obtained from the BDHQ were not comprehensive. For example, deep frying applied to meat and vegetables. Thus, a more detailed questionnaire design is required for further studies.

In conclusion, we investigated the relationship between serum PCB levels and dietary habits in pregnant Japanese women by analysing food items, nutrients, and cooking methods using a BDHQ. The results of the analyses of food items and nutrients suggest that food items such as vegetables, fruits, mushrooms, rice, seaweed, pulses, nuts, and tea, which are rich in dietary fibre, are associated with lower serum levels of PCBs. The results also revealed that cooking methods, such as grilling and deep frying, were associated with lower serum levels of PCBs. However, further investigation must be performed to verify the effects of dietary fibre and cooking methods based on case-control studies, to establish a protocol for healthy dietary habits. The health of future generations may be improved if guidelines for healthy dietary habits are introduced for pregnant women and children from a young age.
